# A comprehensive collection of annotations to interpret sequence variation in human mitochondrial transfer RNAs

**DOI:** 10.1186/s12859-016-1193-4

**Published:** 2016-11-08

**Authors:** Maria Angela Diroma, Paolo Lubisco, Marcella Attimonelli

**Affiliations:** Department of Biosciences, Biotechnologies and Biopharmaceutics, University of Bari, Bari, 70126 Italy

**Keywords:** Mitochondrial genomics, tRNA sequence variation, Annotation and prioritization tools, Bioinformatics analysis, NGS

## Abstract

**Background:**

The abundance of biological data characterizing the genomics era is contributing to a comprehensive understanding of human mitochondrial genetics. Nevertheless, many aspects are still unclear, specifically about the variability of the 22 human mitochondrial transfer RNA (tRNA) genes and their involvement in diseases. The complex enrichment and isolation of tRNAs in vitro leads to an incomplete knowledge of their post-transcriptional modifications and three-dimensional folding, essential for correct tRNA functioning. An accurate annotation of mitochondrial tRNA variants would be definitely useful and appreciated by mitochondrial researchers and clinicians since the most of bioinformatics tools for variant annotation and prioritization available so far cannot shed light on the functional role of tRNA variations.

**Results:**

To this aim, we updated our MToolBox pipeline for mitochondrial DNA analysis of high throughput and Sanger sequencing data by integrating tRNA variant annotations in order to identify and characterize relevant variants not only in protein coding regions, but also in tRNA genes. The annotation step in the pipeline now provides detailed information for variants mapping onto the 22 mitochondrial tRNAs. For each mt-tRNA position along the entire genome, the relative tRNA numbering, tRNA type, cloverleaf secondary domains (loops and stems), mature nucleotide and interactions in the three-dimensional folding were reported. Moreover, pathogenicity predictions for tRNA and rRNA variants were retrieved from the literature and integrated within the annotations provided by MToolBox, both in the stand-alone version and web-based tool at the Mitochondrial Disease Sequence Data Resource (MSeqDR) website. All the information available in the annotation step of MToolBox were exploited to generate custom tracks which can be displayed in the GBrowse instance at MSeqDR website.

**Conclusions:**

To the best of our knowledge, specific data regarding mitochondrial variants in tRNA genes were introduced for the first time in a tool for mitochondrial genome analysis, supporting the interpretation of genetic variants in specific genomic contexts.

**Electronic supplementary material:**

The online version of this article (doi:10.1186/s12859-016-1193-4) contains supplementary material, which is available to authorized users.

## Background

The abundance of biological data characterizing the genomics era is contributing to a comprehensive understanding of human mitochondrial genetics. To date more than 30,000 complete human mitochondrial genomes have been sequenced [1] and lots of tools and databases are publicly available allowing to gather large amounts of information about mitochondrial DNA (mtDNA). Nevertheless many aspects are still unclear, specifically about the 22 human mitochondrial transfer RNAs (mt-tRNA).

Thanks to the “four-way wobble rule” and post transcriptional modifications at the first letters of tRNA anticodons [2], only 22 mt-tRNAs are sufficient in humans, as well as in other mammals, to translate all sense codons into 13 subunits of respiratory chain complexes encoded in each single copy of mtDNA [2]. mt-tRNAs could be considered hot spots of mutations [3]: among more than 600 disease associated mutations compiled to date, about 240 were mapped on mt-tRNA genes [4]. However, it is well known that clinical phenotypes appear only when the mutation load exceeds a certain threshold [5], considering the possible co-existence of different mtDNA genotypes within the same cell, tissue or individual, a condition known as heteroplasmy. Thus, if a mutation in an mt-tRNA gene has no consequences on mtDNA replication or transcription, it may instead affect biogenesis and functioning of tRNAs after their transcription [6]. For instance, post-transcriptional modifications by nuclear-encoded enzymes [7, 8] often occur in key positions for a correct tRNA functioning, including folding and codon-anticodon interaction [6, 9, 10]. As a consequence, the lack of a correct post-transcriptional process could cause pathological effects [11, 12].

Some features are shared among human and other mammalian mt-tRNAs, such as the low number of G–C pairs within stems of the 14 tRNAs encoded by the light DNA strand, due to a strong bias in nucleotide content (A, U and C-rich tRNAs), variable D-loop and T-loop sizes, and lack of conserved and semi-conserved signature motifs [13], thus the difficulties linked to the complex process of human tRNA purification and identification of modified nucleotides are often overpassed through predictions based on bovine models [2].

The availability of information about mt-tRNA genes and variants would support the interpretation of mtDNA variants and improve the understanding of molecular mechanisms of disease. However, most bioinformatics tools for variant annotation and prioritization available so far cannot shed light on the functional role of mt-tRNA variations, often focusing only on characterization of missense variants [14, 15].

To this aim, we updated our MToolBox pipeline [16] for mtDNA analysis of high throughput and Sanger sequencing data by integrating tRNA variants annotations in order to identify relevant variants not only in protein coding regions but also in tRNA genes. Pathogenicity predictions retrieved from the literature were added both for tRNA and rRNA gene variants, when available. These information were also provided as custom tracks which can be visualized in the GBrowse at the Mitochondrial Disease Sequence Data Resource (MSeqDR) website [17], conveniently allowing a deep insight into mitochondrial genomics.

## Methods

### Data collection from known databases, web-based resources and literature

All the information collected in this work and those previously collected and already implemented in the MToolBox pipeline [16], come from several resources and the literature about human mtDNA genomics and variation (Table 1). Nucleotide variability scores calculated by applying *SiteVar* algorithm [18] on 22,691 complete genomes from healthy individuals in the Human Mitochondrial Database, HmtDB (May 2014 update) [19], were reported for each position of the entire human mitochondrial genome; amino acid scores, calculated by *MitVarProt* algorithm [20] on the same dataset, were obtained for coding regions. Conservation scores calculated by *PhyloP* [21] and *PhastCons* [22] algorithms were retrieved from UCSC Genome Browser [23].

**Table 1:** Annotations by MToolBox pipeline

Variant annotation	Status
Locus	Previously provided
HF	Previously provided
CI_lower;CI_upper	Previously provided
RSRS	Previously provided
MHCS	Previously provided
rCRS	Previously provided
Haplogroup	Previously provided
Other Haplogroups	Previously provided
Nt Variability	Updated
Codon Position	Previously provided
Aa Change	Previously provided
Aa variability	Updated
tRNA Annotation	New
Disease Score	Previously provided
RNA predictions	New
MutPred Pred	Previously provided
MutPred Prob	Previously provided
PolyPhen-2 HumDiv Pred	Previously provided
PolyPhen-2 HumDiv Prob	Previously provided
PolyPhen-2 HumVar Pred	Previously provided
PolyPhen-2 HumVar Prob	Previously provided
PANTHER Pred	Previously provided
PANTHER Prob	Previously provided
PhD-SNP Pred	Previously provided
PhD-SNP Prob	Previously provided
SNPs&GO Pred	Previously provided
SNPs&GO Prob	Previously provided
MITOMAP Associated Disease(s)	Updated
MITOMAP Homoplasmy	Updated
MITOMAP Heteroplasmy	Updated
Somatic Mutations	Updated
SM Homoplasmy	Updated
SM Heteroplasmy	Updated
ClinVar	New
OMIM	Updated
dbSNP	Updated
Mamit-tRNA	Previously provided
PhastCons20Way	New
PhyloP20Way	New
AC/AN 1000 Genomes	Previously provided
1000 Genomes Homoplasmy	Previously provided
1000 Genomes Heteroplasmy	Previously provided

All the annotations provided by MToolBox pipeline are shown. In the latest update, new fields, mainly regarding tRNA gene variants, were added for a more accurate variant annotation in analyzed samples: structural information for tRNA variants (“tRNA annotation”), pathogenicity predictions for tRNA and rRNA genes (“RNA predictions”), disease reports in ClinVar database (“ClinVar”), conservation scores (“PhastCons20Way”, “PhyloP20Way”). tRNA annotation, in turn, includes five semi-colon separated annotations: position numbering in tRNA, tRNA type, cloverleaf secondary region, mature nucleotide and involvement of the specific position in tRNA folding (Y for yes or N for no). Moreover, data from HmtDB (“Nt variability”, “Aa variability”), MITOMAP (“MITOMAP Associated Disease(s)”, “MITOMAP Homoplasmy”, “MITOMAP Heteroplasmy”, “Somatic Mutations”, “SM Homoplasmy”, “SM Heteroplasmy”), OMIM links (“OMIM”) and dbSNP identifiers (“dbSNP”) were updated. All the remaining annotations were Previously provided by MToolBox

Somatic mutations and germline variants with reports of disease-associations were available in MITOMAP [4], with corresponding annotation of heteroplasmic/homoplasmic status (July 20, 2015 update of coding and control regions variants; July 29, 2015 update of somatic mutations and RNA genes variants). Other resources were exploited in order to facilitate clinical interpretation of variants, although they are not specialized for mitochondrial genome variant analysis, including OMIM [24], the Online Mendelian Inheritance in Man (August 4, 2015 update), dbSNP [25], a database for short genetic variations (release 144, May 26, 2015), and ClinVar [26], a public archive of reports of human variations and phenotypes reporting annotations of variants found in patient samples (January 21, 2015 update).

Moreover, specific annotations for tRNA variants were gathered from databases, such as Mamit-tRNA [13], mitotRNAdb [27] and MODOMICS [28], as well as from the literature. Specifically, a scoring system developed for 207 variants in tRNA genes considering functional evidence, conservation, frequency and heteroplasmy status in mutations reported in MITOMAP as “pathogenic”, was retrieved [29, 30] and normalized to a 0–1 range (Table 2). Recently published predictions of pathogenicity for DNA variants involving 12S mitochondrial rRNA (mt-rRNA) [31] were considered and adapted, too.

**Table 2:** RNA pathogenicity predictions in MToolBox with corresponding scores

rRNA prediction	rRNA Score	RNA pathogenicity score in MToolBox	tRNA Score	tRNA prediction
Proven pathogenic	5	1.000	20	Definitely pathogenic
		0.950	19	Definitely pathogenic
		0.900	18	Definitely pathogenic
		0.850	17	Definitely pathogenic
Expectedly pathogenic	4	0.800	16	Definitely pathogenic
		0.750	15	Definitely pathogenic
		0.700	14	Definitely pathogenic
		0.650	13	Possibly/definitely pathogenic
Likely pathogenic	3	**0.600**	12	Possibly/definitely pathogenic
		0.550	11	Possibly/definitely pathogenic
		0.500	10	Possibly pathogenic
		0.450	9	Possibly pathogenic
Not enough evidence	2	0.400	8	Possibly pathogenic
		**0.350**	7	Possibly pathogenic
		0.300	6	Neutral
		0.250	5	Neutral
Undetermined	1	0.200	4	Neutral
		0.150	3	Neutral
		0.100	2	Neutral
		0.050	1	Neutral
Unlikely pathogenic	0	0.000	0	Neutral

RNA pathogenicity scores provided by MToolBox pipeline, shown in the central column of the table, derived from two different scoring systems for rRNA and tRNA genes, respectively. Original predictions and scores, reported on the right and the left of MToolBox scores, were retrieved from the literature and normalized to a 0–1 range. Thresholds of 0.600 for rRNA and 0.350 for tRNA sequence variations (in bold) were set according to original scores. Damaging effects could be observed for variants with a score above or equal to the chosen thresholds, while neutral variants should be associated with lower values

### MToolBox

*MToolBox* [16] is a bioinformatics pipeline recently developed for accurate and complete analysis of mitochondrial genome from high throughput sequencing. The tool includes several steps in the data analysis process, such as variant annotation and prioritization by exploiting several annotation resources, such as biological databases [4, 19] and pathogenicity prediction software [32–34], proving to be very useful especially in the characterization of missense variants (Table 1). The pipeline was also developed as a web-based tool, hosted at MSeqDR website [17], a portal recently developed for supporting mitochondrial disease studies by providing both data and user-friendly tools specifically for mtDNA analysis.

### Variant annotators

Both generic and mitochondrial-oriented tools were used for a comparison of variant annotation processes. The command line tools ANNOVAR (version date 2015-03-22) [35], dbNSFP (version 3.0b1a) [14], and SnpEff (version 4.1b) [36], although not specific for mtDNA analysis, were used to provide annotations for three mitochondrial mutations involving genes coding for an rRNA, a tRNA and a protein, respectively. Web-based versions of mit-o-matic [37], MitoBamAnnotator [38] and MitImpact 2.0 [15] tools were also applied to the same mutations to compare their performance in variant annotation.

### GBrowse tracks at MSeqDR website

GBrowse instance at MSeqDR website [17] allows visualization and analysis of variations and other genomics data in a classic genome browser interface by hosting mtDNA specific annotation tracks containing data from some of the major mtDNA genomics resources, such as HmtDB_rCRSvariants and HmtDB_RSRSvariants, provided by our group [17]. Data collection for new tracks generation was manually curated in order to produce tab-delimited text files, then converted in the required format (General Feature Format version 3, GFF3). Variants were reported using the Human Genome Variation Society (HGVS) nomenclature [39].

## Results and discussion

### Annotations for mitochondrial DNA variants in RNA genes by MToolBox pipeline and data update

The MToolBox pipeline [16] was updated and enhanced with specific annotations regarding tRNA genes, introduced for the first time in a tool specific for mtDNA analysis.

**Fig. 1:**
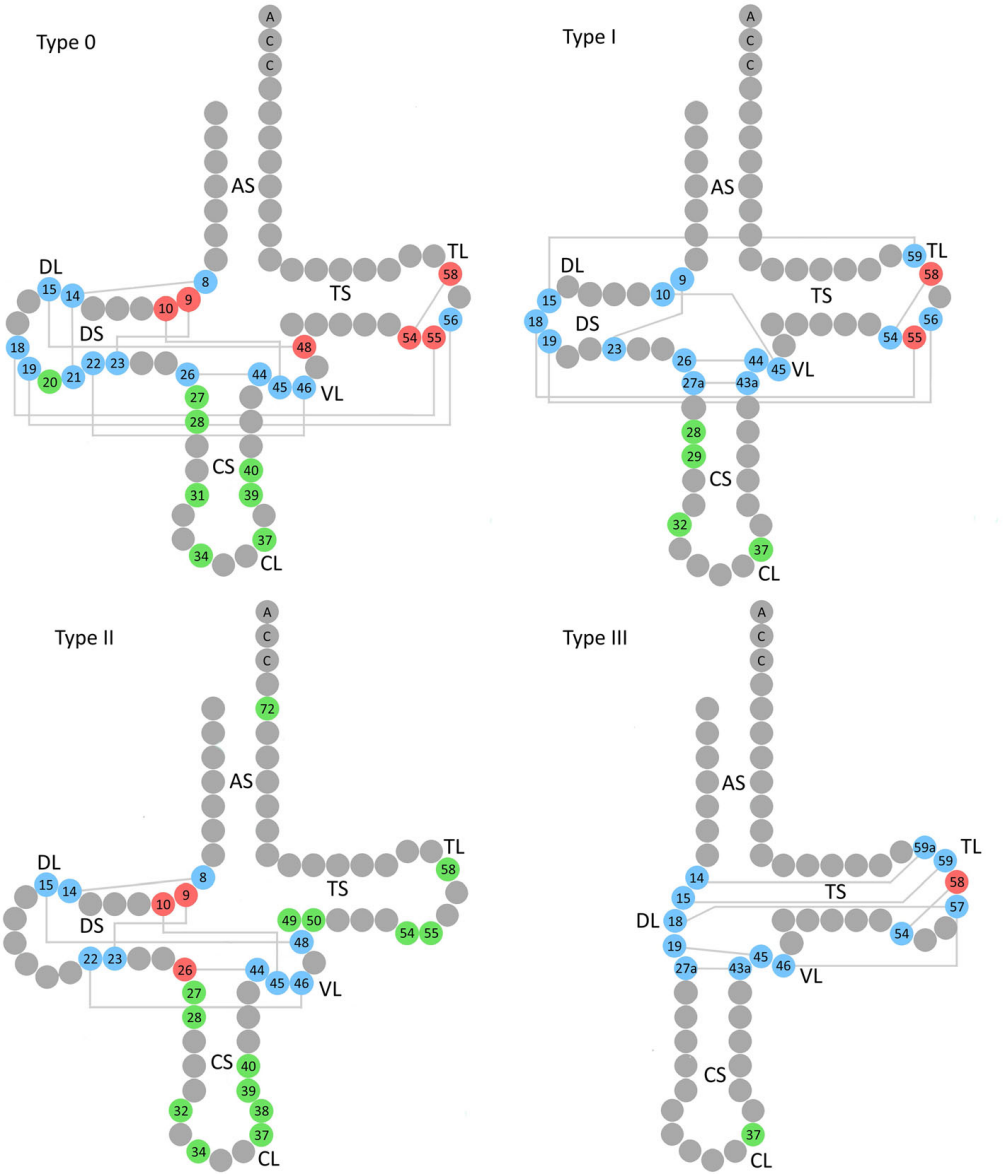
Schematic representation of the four types of human mitochondrial tRNAs. The four types of human mt-tRNAs are shown. *Green circles* represent all the nucleotide positions involved in post-transcriptional modifications in each tRNA. *Blue circles* indicate nucleotide positions involved in tertiary folding with interactions represented by lines. *Red circles* represent nucleotide positions involved in tertiary folding and subject to post-transcriptional modifications. All the stems (A-stem, T-stem, C-stem, D-stem) and loops (T-loop, V-loop, C-loop, D-loop) of cloverleaf secondary regions are also shown

New fields were added in the latest version of the MToolBox pipeline (Table 1): specific annotations for tRNA and rRNA genes, annotations from ClinVar database for disease-associated variants [26] and conservation scores for each site produced by PhyloP [21] and PhastCons [22] algorithms. Specifically, tRNA genes were characterized in each position with reports about tRNA structure including i) position in tRNA, following the Sprinzl standard nomenclature [27]; ii) tRNA type [40]; iii) cloverleaf-shaped secondary structure regions [27]; iv) mature nucleotide [2, 7, 28]; v) involvement of the specific position in tRNA folding [2, 7, 41] (Fig. 1). Each tRNA nucleotide was numbered from 1 to 73, CCA-ending excluded; the anticodon triplet was marked with nucleotides 34 to 36. The tRNA type indicates one of the four possible groups ranking human mt-tRNAs for their structural diversity and different tertiary interactions: type 0, the quasi-canonical cloverleaf structure, with standard D-loop/T-loop interaction; type II, the most common among mt-tRNAs, characterized by loss of D/T-loop interaction; type I and type III, each accounting one single tRNA with an atypical anticodon stem and lack of D-stem, respectively. The annotation of the typical cloverleaf pattern includes abbreviations of four loops (TL-TΨC Loop, VL-Variable Loop, CL-Anticodon Loop, DL-Dihydrouridine Loop), four stems (AS-Acceptor Stem, TS-TΨC Stem, CS-Anticodon Stem, DS-Dihydrouridine Stem), 3′ end (E) and junctions (-).

The mature nucleotide is meant as the nucleotide found in the tRNA molecule after post-transcriptional processes, predicted based on information of bovine and model organisms (bacteria, yeast, nematode) mt-tRNAs, and confirmed in 8 human mt-tRNAs [2, 8]. As a result of our data collection, we annotated 110 residues in the human mt-tRNA set involved in post-transcriptional modifications, with 16 different types of modified nucleotides. All the post-transcriptional modifications in mt-tRNAs and resulting mature nucleotides are listed in Table 3.

**Table 3:** Post-transcriptional modifications in mt-tRNAs

Position	Modified base name	Modomics symbol	Human mt-tRNAs	Bovine mt-tRNAs	Model organisms tRNAs
9	1-methyladenosine	”	Asp, Leu(CUN), Lys, Pro	Ala, Glu, Phe, Gly, His, Asn, Arg, Thr, Val, Trp	
9	1-methylguanosine	K	Ile, Leu(UUR)	Cys, Gln, Tyr	
10	N2-methylguanosine	L	Leu(UUR), Leu(CUN), Lys, Asp		Ala, Phe, Gly, His, Asn, Val, Trp, Tyr
20	dihydrouridine	D	Leu(UUR)		
26	N2-methylguanosine	L			Ala, Glu
26	N2,N2-dimethylguanosine	R	Ile		
27	pseudouridine	P	Asp, Ile, Leu(UUR), Leu(CUN), Lys, Met, Pro	Cys, His	
28	pseudouridine	P	Ile, Lys, Leu(CUN), Pro	Cys, Glu, Asn, Ser(UCN), Tyr	
29	pseudouridine	P			Ser(UCN)
31	pseudouridine	P	Leu(CUN)		
32	3-methylcytidine	’			Ser(UCN), Thr
32	pseudouridine	P	Pro		Cys
34	5-taurinomethyluridine	Ê	Leu(UUR)		Glu, Gln, Trp
34	queuosine	Q	Asp		His, Asn, Tyr
34	5-taurinomethyl-2-thiouridine	Ǝ	Lys	Glu, Gln	
34	5-formylcytidine	>	Met		
37	N6-threonylcarbamoyladenosine	6	Ile, Lys, Ser(AGY)		Asn, Thr
37	N6-isopentenyladenosine	+		Cys, Phe, Ser(UCN), Trp, Tyr	
37	2-methylthio-N6-isopentenyladenosine	*		Phe, Ser(UCN), Trp, Tyr	
37	1-methylguanosine	K	Leu(CUN), Pro	Gln	
38	pseudouridine	P	Pro		
39	pseudouridine	P			Ala, Cys, Phe, Gly, His, Gln, Arg, Tyr
40	pseudouridine	P			Glu, Gln
48	5-methylcytidine	?	Leu(UUR)		
49	5-methylcytidine	?			Glu
50	pseudouridine	P	Met		
54	5-methyluridine	T	Leu(UUR), Pro		
55	pseudouridine	P	Leu(UUR), Pro		Glu, Gln, Ser(UCN), Tyr
58	1-methyladenosine	”	Ser(AGY), Leu(UUR)	Cys, Ser(UCN)	Glu
72	5-methylcytidine	?			Thr

All the post-transcriptional modifications confirmed or predicted in human mt-tRNAs are listed. The full name of modifications, Modomics symbols and positions affected are shown for each tRNA species. Modifications reported include those confirmed by crystallographic data in eight human mt-tRNAs, those predicted using bovine model, which has similar structure and sequence in mt-tRNAs, and those predicted based on model organisms, such as bacteria, yeast and nematode

Indication of the involvement of a specific residue in tRNA folding could be now recovered through variant annotation by our updated version of MToolBox. The three-dimensional structure of mt-tRNA has a typical L-shape, due to the molecule folding back in itself forming two double helix segments through base pairing between T and D loop. Triplet interactions also occur in position 10-25-45, 9-23-12 and 13-22-46 in order to increase stability [7]. The strength of folding is also affected by base stacking interactions, interesting almost all the nucleotides [42].

As expected, we observed a relatively low frequency of disease associated mutations within the anticodon triplet (11/394 mutations) since its high conservation is required for a correct recognition of the messanger RNA. Specifically, position 36, corresponding to the third base within anticodon, is more subject to pathogenic mutations (7/11). Moreover we observed a quite homogeneous distribution of mutations with a deleterious effect in other tRNA regions, in line with an almost consistent involvement of all the regions in the three-dimensional folding.

Fortynine variants in rRNA genes [31] and 207 variants in tRNA genes [29, 30] were retrieved from the literature as validated mutations, hence inserted within the annotation mechanism used by MToolBox and integrated with pathogenicity predictions and scores. Original scores were normalized to a 0–1 range, with derived thresholds of 0.600 and 0.350 for rRNA and tRNA sequence variations, respectively (Table 2). Damaging effects could be observed for variants with a score above or equal to the chosen thresholds, while neutral variants should be associated with lower values.

Finally, several annotations previously collected [16] were accurately revised to provide users the most possible up-to-date pipeline for mitochondrial genome analysis, including updated variability data from HmtDB database [19], dbSNP identifiers [25], OMIM links to known variants [24], novel disease associated variants and somatic mutations reported in MITOMAP [4] (Table 1).

All the updates in MToolBox are available both in the command line version [43] and in the web-based resource at MSeqDR website [44]. New options to better manage input files are described in the readme file in the package. Moreover a summary is now produced reporting all the parameters chosen for the analysis and some basic statistics.

### Annotation/prioritization tools comparison

In recent years lots of tools for variant prioritization were produced in order to help clinicians and researchers to recognize a few relevant mutations among the huge amount of variations detectable by NGS technologies. However, the annotation and prioritization processes carried out by these tools are often focused on missense variant characterization by providing pathogenicity predictions, dbSNP identifiers, frequency in known datasets such as the 1000 Genomes, conservation scores and region annotations (see Additional file 1). Among the most popular tools for variant prioritization, ANNOVAR [35], SnpEff [36] and dbNSFP [14] are commonly used both for nuclear DNA and mtDNA variations. Moreover mitochondrial-oriented tools have been recently developed, such as mit-o-matic [37], MitImpact [15] and MitoBamAnnotator [38] to ensure appropriate annotations mindful of mitochondrial genetics peculiarities, such as heteroplasmy. A comparison was performed among the aforementioned tools, showing pros and cons of each of them (Additional file 1). A few generic annotations regarding mt-tRNA variants were provided by some of the tested tools, while the MToolBox pipeline showed a wide range of annotations proving to be useful for any variant evaluation and not only missense variants (Table 4). Moreover, several input file formats can be used by MToolBox, proving a great efficiency for both high throughput sequencing and traditional FASTA data. Last but not least, the web-based version of the tool [44] ensures large usability also by non-expert users interested in mitochondrial genome analysis.

**Table 4:** Variant annotators comparison for a tRNA gene mutation

MToolBox Annotation	Example
Locus	MT-TM
RSRS	Yes
MHCS	Yes
rCRS	Yes
Haplogroup	
Other Haplogroups	
Nt Variability	0.00E+00
tRNA Annotation	53;II;TS;G;N
Patho-prediction RNA coding genes	0.65
MITOMAP Associated Disease(s)	Myopathy
MITOMAP Homoplasmy	N
MITOMAP Heteroplasmy	Y
Mamit-tRNA	http://mamit-trna.u-strasbg.fr/mutations.asp?idAA=19
PhastCons20Way	0.889764
PhyloP20Way	0.797921
SnpEff Annotation	Example
Annotation	non_coding_exon_variant
Annotation_Impact	MODIFIER
Gene_Name	MT-TM
Gene_ID	ENSG00000210112
Feature_Type	Transcript
Feature_ID	ENST00000387377
Transcript_BioType	Mt_tRNA
Rank	1/1
HGVS.c	n.49G>A
MitoBamAnnotator	Example
pos	4450
ref	G
from	G
to	A
gene	MT-TM
coding for	tRNA
coding region	0
is mutated	1
triplet position	NA
mutation type	NA
has_overlap	0
CI	NA
mit-o-matic	Example
Variant	4450
Ref allele	G
Alt allele	A
Gene	MT-TM
Disease	Myopathy

Among tools providing annotations for a specific variant in a tRNA gene (*m.4450G>A*) chosen for its potential damaging effect, MToolBox showed the widest range of useful features provided in the final annotation step allowing users to prioritize the variant. Empty fields were omitted. Tested tools which do not provide annotations for tRNA variants were not reported

### Mitochondrial variations tracks at MSeqDR

**Fig. 2:**
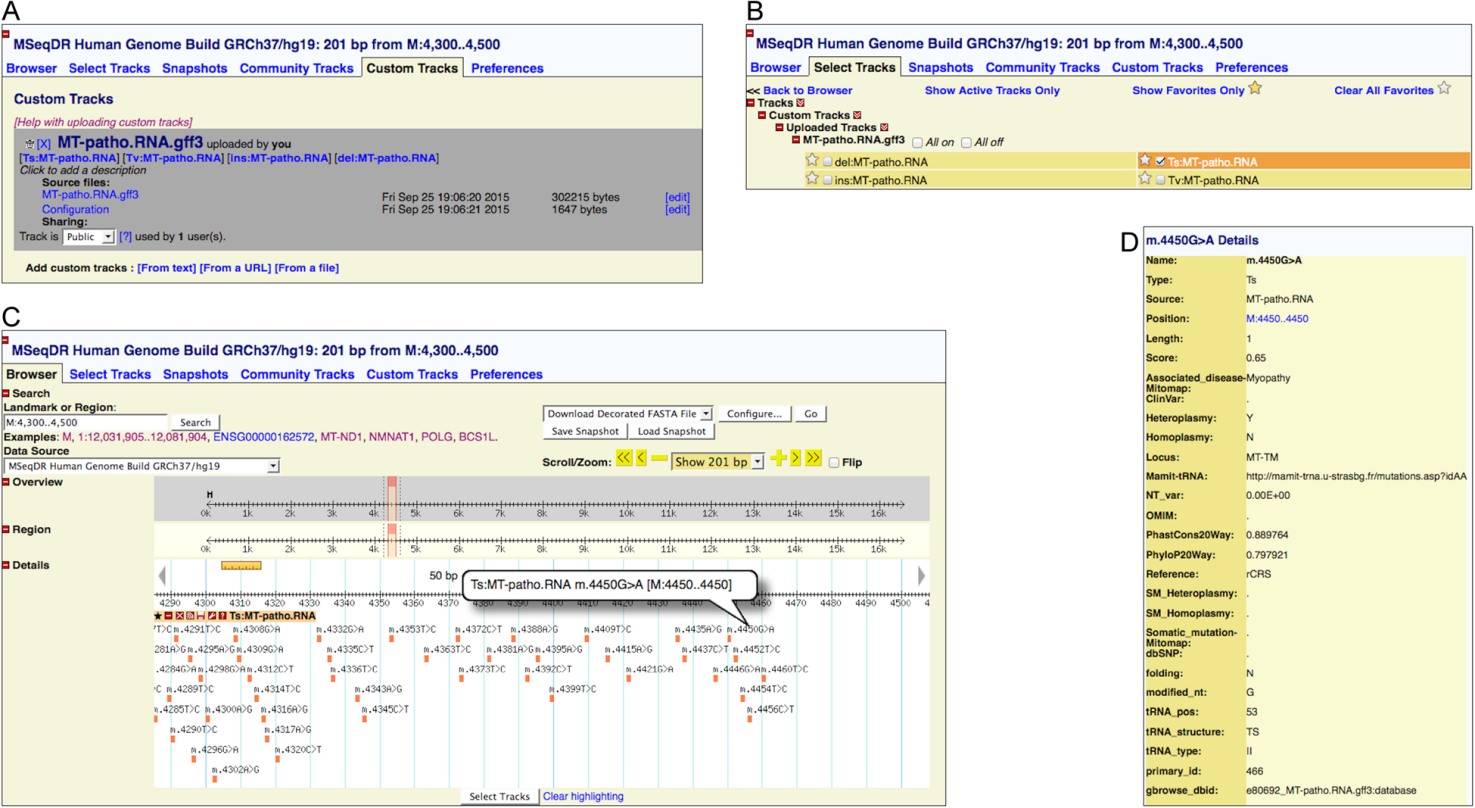
Overview of the usage of mitochondrial tracks at MSeqDR GBrowse. MSeqDR website provides access to a GBrowse useful to visualize genomics data. Users can upload the four tracks generated in this work in the “Custom Tracks” section of the browser (**a**). For the sake of simplicity, the only “MT-patho.RNA” track is here shown, including data about pathogenic variants in mt-tRNA and mt-rRNA genes. The custom track can be selected, totally or partially (only transitions, transversions, insertions or deletions, **b**) and then visualized in the browser (**c**) where users can search for a specific genomic region of interest. Eventually, detailed information can be shown by clicking on a specific variant site (**d**)

In order to facilitate the interpretation of genetic variants in a specific genomic context, four different custom tracks were produced in GFF3 file format displayable at MSeqDR GBrowse [45] (Fig. 2). The tracks included all the data used for the annotation step carried out by the MToolBox pipeline, providing users the possibility to analyze only variants or genomic positions with no need to provide input files. A track previously provided, called “Mitochondrial Pathogenicity Predictions” [17], was updated and split into two different tracks, “MT-patho.CDS” and “MT-patho.STOP” tracks. The first collects all the 24,202 possible non-synonymous variants within the 13 human mitochondrial protein encoding genes, identified using mtDNA-GeneSyn software [46]. Predictions and probabilities of pathogenicity were produced using five different software [16] and an overall disease score was also provided [47].

The second track collects all the 1740 possible stop-gain and 77 possible stop-loss mutations, which could be damaging in the generation of the 13 human mitochondrial proteins.

The third track (“MT-patho.RNA”) is useful to show all the information currently available about pathogenicity of 392 variants in tRNA and 337 in rRNA genes, while the fourth track (“MT-RNA”) includes generic annotations reported for all the 1505 positions in genes encoding tRNAs and 2513 positions in genes encoding rRNAs, respectively. All the tracks were produced using the revised Cambridge Reference Sequence, rCRS (GenBank: J01415.2), as reference sequence.

Additional information from MITOMAP [4], ClinVar [26], Mamit-tRNA [13] dbSNP [25] and OMIM [24] databases were shown, when available, for all the four tracks, as well as variability data from HmtDB database [19] and conservation scores from UCSC Genome Browser [21, 22].

The tracks, can be uploaded in the “Custom Tracks” section of the MSeqDR website, selected, totally or partially (only transitions, transversions, insertions or deletions) and visualized in the GBrowse (Fig. 2).

## Conclusions

To the best of our knowledge, specific data regarding mitochondrial variants in tRNA genes were introduced for the first time in a tool for mitochondrial genome analysis and then reported in custom tracks, which could be displayed at MSeqDR GBrowse. The availability of such data could be useful to support the interpretation of genetic variants in specific genomic contexts.

## Additional file

Additional file 1: Variant annotation by 7 different tools. All the annotations provided by MToolBox, ANNOVAR, SnpEff, dbNSFP, MitImpact 2.0, MitoBamAnnotator and mit-o-matic are shown. Three variants were considered (*m.879T>C*, *m.3436G>C*, *m.4450G>A*), one for an rRNA gene (*MT-RNR1*), one for a tRNA gene (*MT-TM*) and one for a protein coding gene (*MT-ND1*). ANNOVAR and SnpEff tools use dbNSFP databases. Generally, all the tools provided an accurate annotation for the missense variant, although we were not able to obtain any information by mit-o-matic web-based software. MToolBox provided the most complete annotation for non protein coding regions. (XLSX 44 kb)

## Abbreviations

AS: Acceptor stem
CL: Anticodon loop
CS: Anticodon stem
DL: Dihydrouridine loop
DS: Dihydrouridine Stem
GFF3: General feature format version 3
HGVS: Human genome variation society
HmtDB: Human mitochondrial database
MSeqDR: Mitochondrial disease sequence data resource
mtDNA: Mitochondrial DNA
mt-rRNA: Mitochondrial ribosomal RNA
mt-tRNA: Mitochondrial transfer RNA
rCRS: Revised Cambridge Reference Sequence
TL: TΨC Loop
TS: TΨC stem
VL: Variable loop
